# Bioinspired phototransistor with tunable sensitivity for low-contrast target detection

**DOI:** 10.1038/s41377-025-02051-1

**Published:** 2026-01-01

**Authors:** Ruyue Han, Dayu Jia, Bo Li, Shun Feng, Guoteng Zhang, Yun Sun, Zheng Han, Chi Liu, Hui-Ming Cheng, Dong-Ming Sun

**Affiliations:** 1 Shenyang National Laboratory for Materials Science, Institute of Metal Research, Chinese Academy of Sciences, Shenyang, China; 2 School of Materials Science and Engineering, University of Science and Technology of China, Shenyang, China; 3 School of Information Institution, Liaoning University, Shenyang, China; 4 State Key Laboratory of Quantum Optics and Quantum Optics Devices, Institute of Opto-Electronics, Shanxi University, Taiyuan, China; 5 Collaborative Innovation Center of Extreme Optics, Shanxi University, Taiyuan, China; 6 Liaoning Academy of Materials, Shenyang, China; 7 Faculty of Materials Science and Engineering/Institute of Technology for Carbon Neutrality, Shenzhen Institute of Advanced Technology, Chinese Academy of Sciences, Shenzhen, China

**Keywords:** Imaging and sensing, Photonic devices

## Abstract

Accurate recognition of low-contrast targets in complex visual environments is essential for advanced intelligent machine vision systems. Conventional photodetectors often suffer from a weak photoresponse and a linear dependence of photocurrent on light intensity, which restricts their ability to capture low-contrast features and makes them susceptible to noise. Inspired by the adaptive mechanisms of the human visual system, we present a molybdenum disulfide (MoS_2_) phototransistor with tunable sensitivity, in which the gate stack incorporates a heterostructure diode—composed of O-plasma-treated MoS_2_ and pristine MoS_2_—that serves as the photosensitive layer. This configuration enables light-intensity-dependent modulation of the diode’s conductance, which dynamically in turn alters the voltage distribution across the gate dielectric and transistor channel, leading to a significant photoresponse. By modulating the gate voltage, the light response range can be finely tuned, maintaining high sensitivity to low-contrast targets while suppressing noise interference. Compared to conventional photodetectors, the proposed device achieves a 1000-fold improvement in sensitivity for low-contrast signal detection and exhibits significantly enhanced noise immunity. The intelligent machine vision system built on this device demonstrates exceptional performance in detecting low-contrast targets, underscoring its promise for next-generation machine vision applications.

## Abstract (web summary)

Inspired by human vision, a MoS_2_ phototransistor with gate-tunable sensitivity enables precise detection of low-contrast targets and strong noise suppression for next-generation intelligent machine vision systems.

## Graphical abstract

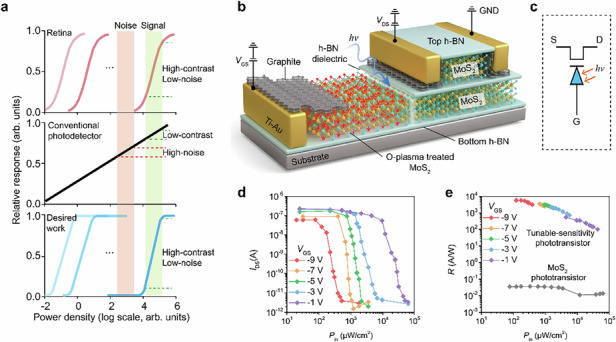

## Introduction

Intelligent machine vision applications, such as precision guidance, smart surveillance, and early warning systems, demand sensors that can generate significant and substantial electrical responses to faint variations in light intensity under complex lighting conditions^1–4^. However, conventional photodetectors based on photodiodes and phototransistors mainly rely on photogenerated carriers within junctions or channels to produce photocurrents^5^. Due to their weak photoresponse and the linear relationship between photocurrent and light intensity, these devices are inherently limited in their ability to capture the features of targets in low-contrast scenes and are highly susceptible to optical noise. To address these limitations, neuromorphic vision devices often employ long exposure times or repetitive imaging to enhance target contrast and suppress noise^6–9^. Nevertheless, such strategies struggle to accommodate rapidly changing scenes. Dynamic vision sensors (e.g., those proposed by Yang et al.) leverage combinations of multiple photodetectors and complex circuit architectures to extract image edge features^10^. However, these approaches unavoidably increase system complexity. Consequently, designing efficient and compact photodetectors capable of detecting low-contrast targets in challenging environments remains a significant challenge.

**Fig. 1:**
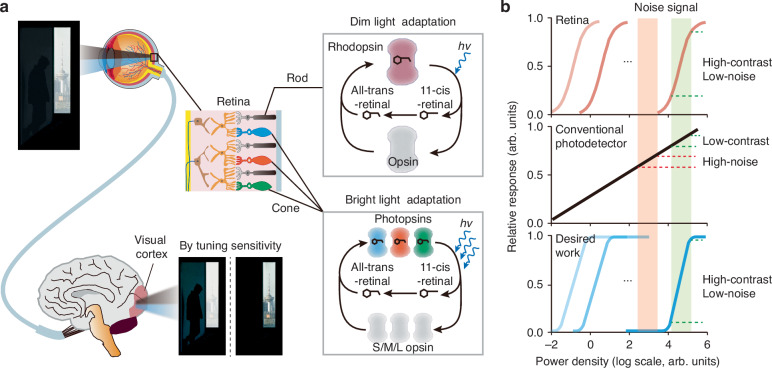
**Bioinspired photodetector with tunable sensitivity**. **a** The retina achieves efficient visual perception under varying illumination conditions through light adaptation. Rod and cone cells adjust their sensitivity via the synthesis and degradation of rhodopsin and photopsins. With increasing light intensity, retinal in rhodopsin and photopsins undergoes isomerization from 11-cis to all-trans, leading to the separation of retinal from opsins and the formation of inactive opsins. Cone cells express S/M/L opsins, which respond to short, medium, and long wavelength light, respectively. **b** Comparison of light responses among the retina, conventional photodetectors, and the bioinspired photodetector. Both the retina and the bioinspired photodetector enhance contrast and filter noise, whereas conventional photodetectors, limited by fixed sensitivity and a linear relationship between photocurrent and light intensity, struggle to effectively detect low-contrast targets

In the human visual system, the mechanism of light adaptation enables visual perception across complex lighting conditions (Fig. 1a). Specifically, cone cells and rod cells in the retina are responsible for detecting bright and dim light, respectively, and their sensitivities are dynamically tuned according to ambient light intensity^11,12^. Photoreceptive proteins, such as rhodopsin and photopsin, undergo synthesis or decomposition in response to changes in light intensity (Fig. 1a). This adaptive mechanism allows the human eye to achieve a global dynamic range exceeding 160 dB (a light intensity difference of approximately 10^8^ times) while maintaining a local dynamic range of 40 dB (a light intensity difference of approximately 10^2^ times) under specific conditions (Fig. 1b)^13–15^. By focusing on relative differences in light intensity rather than absolute illumination levels, the retina excels in recognizing low-contrast targets. Furthermore, it suppresses irrelevant noise signals outside specific intensity ranges, enhancing feature detection in complex dynamic environments. In contrast, conventional photodetectors lack such adaptive sensitivity, limiting their effectiveness in noise suppression and low-contrast detection (Fig. 1b).

Mimicking the adaptive sensitivity of the human eye represents a promising strategy for achieving efficient low-contrast target detection. Compared to bulk materials, two-dimensional materials possess unique electrical and optoelectronic properties^16–18^, enabling the construction of van der Waals heterostructures with high detection sensitivity^19–23^.

Here, we report a molybdenum disulfide (MoS_2_) phototransistor with tunable sensitivity, wherein the key innovation lies in the integration of an oxygen plasma treated (O-plasma-treated) MoS_2_/MoS_2_ heterostructure diode as the photosensitive layer within the device gate. The dynamic adjustment of the diode’s electrical conductivity with light intensity alters the voltage distribution across the dielectric layer and channel of the transistor, resulting in a significant change in photoresponse. By modulating the gate voltage, the phototransistor achieves precise control over the photoresponse range, enabling high-sensitivity detection of low-contrast targets. Compared to conventional photodetectors, this device exhibits over 1000-fold improvement in detection sensitivity and demonstrates exceptional noise tolerance.

## Results

### Device design and characteristics

**Fig. 2:**
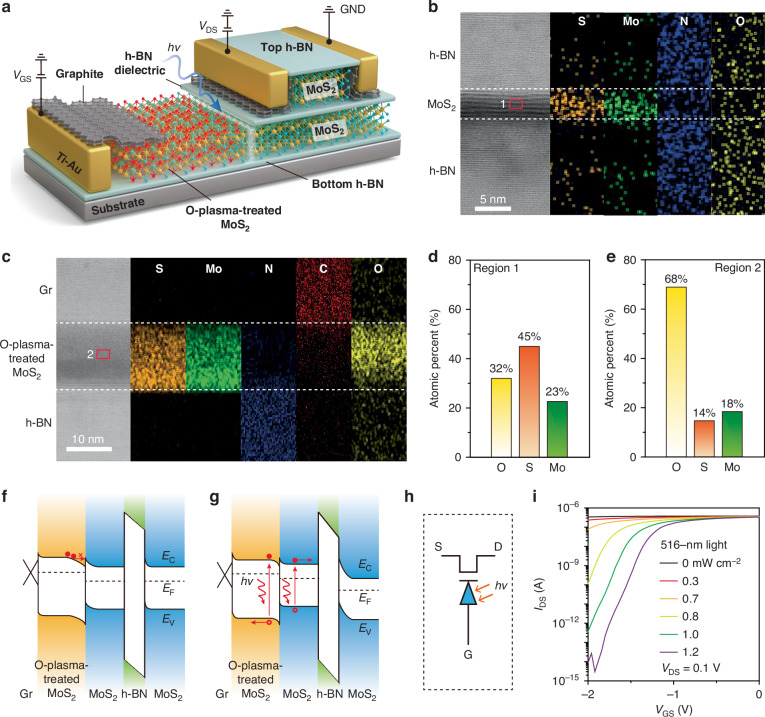
**Phototransistor with tunable sensitivity**. **a** Schematic of the phototransistor, consisting of a MoS_2_ FET integrated with a photodiode. h-BN serves as both the dielectric and protective layer, while graphite is used as the contact and gate electrode. **b** Cross-section HRTEM image of the h-BN/MoS_2_/h-BN heterojunction and elemental maps of S, Mo, N, O for the heterojunction. **c** Cross-section HRTEM image of the Gr/ O-plasma-treated MoS_2_/h-BN heterojunction and elemental maps of S, Mo, N, C, O for the heterojunction. **d**, **e** O, S, and Mo atomic ratio for pristine and O-plasma-treated MoS_2_ samples. **f** Band structure of the device in the dark. **g** Band structure of the device under illumination. **h** Device symbol showing a diode inserted in the gate stack of the FET. **i** Transfer characteristics (*I*_DS_−*V*_GS_) of the tunable-sensitivity phototransistor in dark and under varying 516-nm light intensities at *V*_DS_ = 0.1 V

A tunable-sensitivity phototransistor was designed and fabricated using a layer-transfer method (Methods, Fig. S1). The device is based on a MoS_2_ field effect transistor (FET) with an O-plasma-treated MoS_2_/MoS_2_ diode inserted within the gate stack. Specifically, graphite was used to form the Ohmic contact^24^, MoS_2_ as the channel, hexagonal boron nitride (h-BN) as the dielectric layer, and top and bottom protective layers. An O-plasma-treated MoS_2_/MoS_2_ diode^25–27^was embedded between the h-BN dielectric layer and the bottom protective layer, serving as the photosensitive structure (Figs. 2a and S2). To elucidate the effects of O-plasma treatment on the composition and structure of MoS_2_, comprehensive characterizations of the MoS_2_ samples before and after treatment were performed. Cross-sectional high-resolution transmission electron microscopy (HRTEM) of the h-BN/MoS_2_/h-BN heterostructure reveals a well-defined layered structure. Mo and S are confined within the MoS_2_ layer, N aligns with h-BN, and oxygen is uniformly adsorbed on the cross-section (Fig. 2b). In contrast, the upper layer of the O-plasma-treated MoS_2_ becomes amorphous, confirmed by the presence of Mo, S, and O in energy dispersive X-ray spectroscopy (EDS) mapping (Fig. 2c). For pristine MoS_2_, the S/Mo atomic ratio is approximately 1.95, consistent with the X-ray photoelectron spectroscopy (XPS) results (Figs. 2d and S3), and the oxygen content is about 32%, mainly originating from adsorbed oxygen (Fig. S3). After oxygen plasma treatment, the total oxygen content in the sample increases significantly to 68%, with about 36% of the oxygen incorporated as lattice oxygen. Meanwhile, the Mo and S contents are around 18% and 14%, respectively, corresponding to an approximate molecular formula of MoO_2_S (Fig. 2e). Furthermore, plan-view HRTEM (Fig. S4), EDS (Fig. S4), and Raman characterizations (Fig. S5) further confirm the structural and compositional transition induced by O-plasma treatment.

XPS valence band spectra reveal that the valence band maximum shifts from ~0.8 eV below the Fermi level in pristine MoS₂ to ~1.2 eV after plasma treatment (Fig. S6). Ultraviolet photoelectron spectroscopy measurements further show an increase in work function from ~4.3 eV for pristine MoS_2_ to ~4.7 eV O-plasma-treated MoS_2_^28^. Theoretical calculations indicate that the bandgap widens with oxygen incorporation, increasing from 1.56 eV (MoS_2_) to 1.8 eV (MoO_2_S)^29^. Based on these experimental and theoretical results, schematic band diagrams for pristine and O-plasma-treated MoS_2_ were constructed (Fig. S7). The band alignment suggests the formation of a MoS_2_/O-plasma-treated MoS_2_ n/n^−^ junction, which we experimentally validated by constructing both in-plane and vertical heterojunctions (Figs. S8 and S9). The working principle of the phototransistor with tunable sensitivity was shown in Fig. 2f, g. Under a negative gate bias (*V*_GS_), the O-plasma-treated MoS_2_/MoS_2_ heterojunction is reverse-biased in the dark, causing most of the gate voltage to drop across the junction and keeping the MoS_2_ channel conductive (Figs. 2f and S10). Upon illumination, photogenerated carriers reduce the junction resistance, redistributing the gate voltage such that a larger portion is applied across the h-BN dielectric and MoS_2_ channel, thereby depleting the channel and turning the transistor off (Figs. 2g and S11). As the gate voltage becomes more negative, the voltage across the MoS_2_ channel increases further, enabling the device to shut off at lower light intensities, thus realizing tunable detection sensitivity. A new device symbol representing this structure is proposed (Fig. 2h).

Figure 2i shows the photoresponse behavior of the phototransistor. When the gate voltage is −2 V, the device shows only a slight current change at light intensities below 0.7 mW cm^−2^. However, within the light intensity range of 0.7–1.2 mW cm^−2^, the device exhibits a current change of nearly 10^7^ times. In contrast, the current change in the O-plasma-treated MoS_2_/MoS_2_ diode is only 1.6 times (Fig. S12). These demonstrate that our transistor can generate a non-linear relationship between photocurrent and light intensity, mimicking the retina system. To exclude the contribution of the MoS_2_ channel to the photocurrent, we conducted position-dependent illumination experiments, confirming that the dominant photoresponse originates from the O-plasma-treated MoS_2_/MoS_2_ heterojunction (Fig. S13). In addition, the transistor behavior under light is different under different *V*_GS_, demonstrating a tunable photosensitivity by varying *V*_GS_.

### Tunable-sensitivity optoelectronic characteristics

**Fig. 3:**
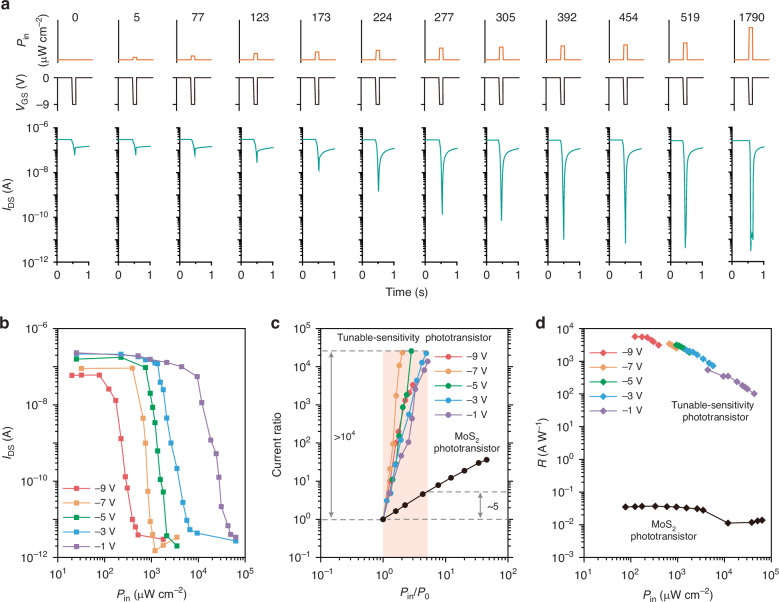
**Optoelectronic performance of the tunable-sensitivity phototransistor**. **a** Photo-response behavior of the device under 516-nm light pulses (pulse width: 100 ms) at varying light intensity (*P*_in_) and gate voltage (*V*_GS_) pulse from 0 to −9 V (pulse width: 100 ms). *V*_DS_ = 0.1 V. **b** Dependence of *I*_DS_ on *P*_in_ under 516-nm light at different *V*_GS_. **c** Variation of the current ratio (tunable-sensitivity phototransistor: *I*_0_/*I*_light_; conventional phototransistor: *I*_light_/*I*_0_) as a function of the light intensity ratio (*P*_in_/*P*_0_) at different *V*_GS_, where *P*_0_ represents the minimum detectable light intensity, and *I*_0_ is the corresponding current. **d** Comparison of responsivity (*R* = |*I*_Light_−*I*_Dark_|/*P*_in_) of the tunable-sensitivity phototransistor and conventional phototransistor under different *P*_in_

Figure 3 shows the detailed optoelectronic performance of the tunable-sensitivity phototransistor. When a *V*_GS_ of −9 V and a 100 ms light pulse are applied simultaneously, the device shows no obvious photoresponse when the light intensity is below 77 μW cm^−2^. However, when the light intensity further increases, the transistor current decreases abruptly. Once the light intensity surpasses 454 μW cm^−2^, the photoresponse of the device reaches saturation (Fig. 3a, b). This non-linear characteristic enables the device to effectively filter both strong-light and weak-light noise. In addition, by adjusting *V*_GS_, the device’s response range to light intensity can be tuned. For instance, when *V*_GS_ = −7 V, the device exhibits a light response range of 392–1061 μW cm^−2^; whereas, at −5 V, the response range is adjusted to 748–2122 μW cm^−2^. Overall, the device can precisely distinguish light intensities within the range of 77–50000 μW cm^−2^ by changing *V*_GS_ (Figs. 3b and S14).

To further demonstrate the device’s ability to detect small changes in light intensity, we extracted the relationship between the current ratio and the light intensity ratio (*P*_in_/*P*_0_) at different *V*_GS_ (Fig. 3c). When the light intensity changes by 3–5 times, the current ratio of the tunable-sensitivity phototransistor exceeds 10^4^. In contrast, the current ratio of the conventional MoS_2_ phototransistor is about 5. This indicates that the tunable-sensitivity phototransistor has more than 1000 times higher capability in detecting small changes in light intensity compared to conventional photodetectors, and also outperforms previously reported gate-tunable phototransistors designed for contrast enhancement^8–10,30–35^ (Table S1). The tunable-sensitivity phototransistor also demonstrates a significantly higher responsivity compared to the conventional MoS_2_ phototransistor (Fig. 3d). Notably, as the light intensity increases, the responsivity of the tunable-sensitivity phototransistor gradually decreases, which is similar to that of the human retina (Fig. 3d). In contrast, the conventional MoS_2_ phototransistor lacked this characteristic (Figs. 3d and S15).

### Performance of the tunable-sensitivity phototransistor array

**Fig. 4:**
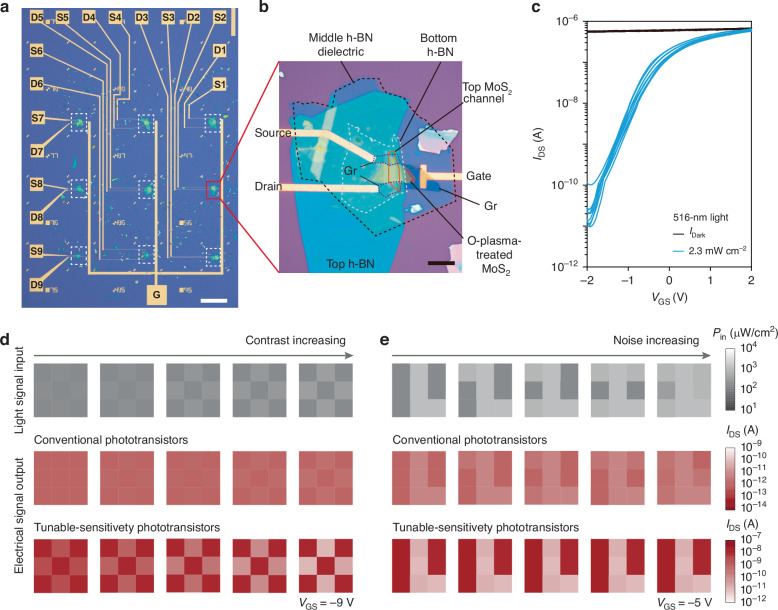
**Performance of tunable-sensitivity phototransistor array**. **a** Optical image of a 3 × 3 phototransistor array (scale bar: 200 μm). **b** Magnified image of an individual sensor unit (scale bar: 10 μm). **c** *I*_DS_−*V*_GS_ curves of the 9 phototransistors in dark and under 516-nm light at *V*_DS_ = 0.1 V. **d**, **e** Comparison of input and output images, demonstrating the advantages of tunable-sensitivity phototransistor array in contrast enhancement (**d**) and noise filtering (**e**) compared with the conventional phototransistor arrays

Figure 4a, b show a 3 × 3 photo sensor array based on tunable-sensitivity phototransistors, exhibiting good uniformity across all 9 transistors both in dark and under light conditions (Figs. 4c and S16). To demonstrate the ability to detect low-contrast targets, we input five sets of low-contrast signals of pattern “O” into both the conventional phototransistor array and the tunable-sensitivity phototransistor array. The light-to-background intensity ratio of pattern “O” ranges from 1.2 to 2.1. For the conventional phototransistor array, the output current ratio is only 1.3 when the light-to-background intensity ratio is 1.2, and increases modestly to 1.7 at a ratio of 2.1, which is insufficient to produce a clear image. In contrast, the tunable-sensitivity phototransistor array achieves a significantly higher current ratio of 3.4 under the same low-contrast condition (intensity ratio of 1.2), and up to 470 at an intensity ratio of 2.1, successfully enabling the recognition of a distinct “O” pattern. This demonstrates the superior performance of the tunable-sensitivity phototransistor in low-contrast target detection (Fig. 4d). Additionally, the developed array exhibited outstanding noise filtering capability. When an image “L” with increasing surrounding noise is input to the conventional phototransistor array, the output image “L” gradually becomes blurred as the noise intensity increases, due to the wide response range of the conventional detector. In contrast, under a gate voltage of −5 V, the tunable-sensitivity phototransistor array is selectively responsive to light intensities in the range of 748–2122 μW cm^−2^, making it immune to out-of-range light noise and allowing it to consistently produce a clear image (Fig. 4e).

### Highly robust target recognition

**Fig. 5:**
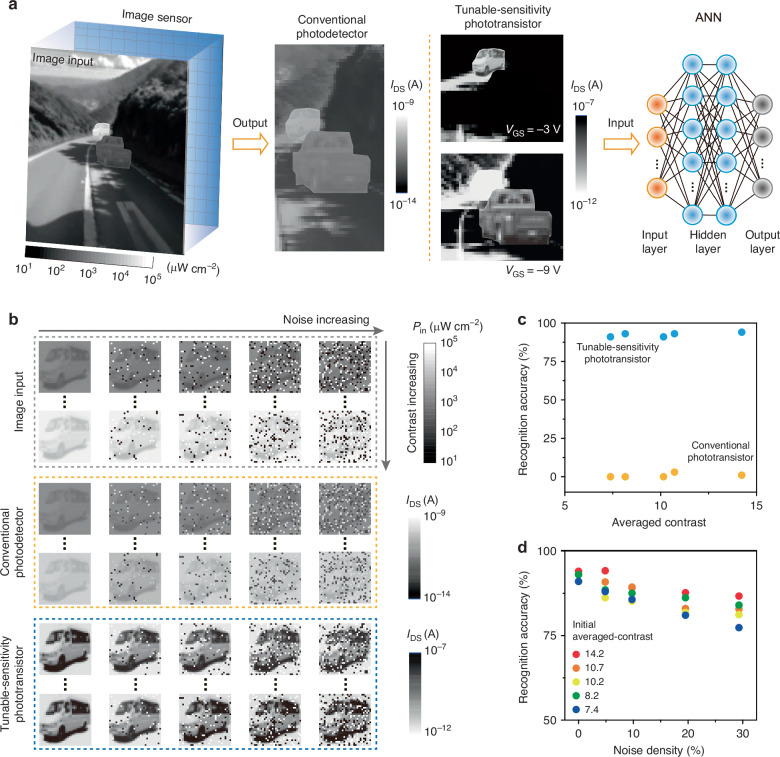
**Robust recognition of low-contrast targets**. **a** Schematic of an intelligent machine vision system integrating tunable-sensitivity phototransistor arrays or conventional sensor arrays with an ANN. The tunable-sensitivity phototransistor enables precise extraction of low-contrast targets. **b** Imaging comparison using conventional phototransistor arrays and tunable-sensitivity phototransistor arrays. **c** Recognition accuracy of tunable-sensitivity phototransistors and conventional phototransistors after 100 training epochs at different average contrast conditions. **d** Tunable-sensitivity phototransistors exhibit high robustness in recognition under salt-and-pepper noise interference

The developed tunable-sensitivity phototransistor enables highly robust target recognition, demonstrated by integrating phototransistor arrays with an artificial neural network (ANN)-based intelligent machine vision system (Fig. 5a). An ANN model was employed to perform classification and recognition based on the image data generated by the phototransistor array. The ANN architecture consisted of an input layer, two hidden layers (the first with 128 neurons and the second with 64 neurons), and an output layer. The rectified linear unit (ReLU) was used as the activation function, and the cross-entropy function was used as the loss function. Conventional photodetectors capture all optical signals in the scene, making it challenging to distinguish low-contrast vehicle targets. On the other hand, by adjusting the gate voltage, our tunable-sensitivity phototransistor responds only to light signals within specific intensity ranges (Table S2). This capability allows accurate recognition of low-contrast vehicle targets in complex lighting conditions, whether under dim or bright lighting conditions, while effectively filtering out noise signals that interfere with target recognition.

For the low-contrast object recognition task, we selected 500 “bus” images from the CIFAR-100 dataset as positive samples, which were not included in the testing set. An additional 1000 images of other vehicles (e.g., motorcycles, bicycles, etc.) were selected as negative samples to establish a binary classification task (Methods). To evaluate the system’s ability to recognize low-contrast targets, we used 500 images of buses, each with a resolution of 32 × 32 pixels, that were not included in the training set as the test dataset. The test dataset consists of five sub-datasets, each containing 100 images, with the average image contrast gradually decreasing across the datasets (Figs. 5b and S17). Figure 5c shows the recognition accuracy after 100 training epochs for each dataset. The machine vision system based on the tunable-sensitivity phototransistor achieved an accuracy exceeding 90%. In contrast, the system based on conventional photodetectors achieved an accuracy close to zero (Fig. 5c).

To evaluate the reliability of the tunable-sensitivity phototransistor-based machine vision system under complex lighting conditions, we introduced salt-and-pepper noise with densities of 5%, 10%, 20% and 30% to 500 test images (Figs. 5b and S18). These 2000 noisy images were then input into the system for recognition. Even with a noise density of 30%, the system’s image recognition accuracy remained around 80% (Fig. 5d). These results highlight the superior robustness of our tunable-sensitivity phototransistor in low-contrast imaging, making it highly suitable for imaging tasks in complex lighting conditions.

## Discussion

Inspired by the adaptive mechanism of the human retina, we developed a tunable-sensitivity MoS_2_ phototransistor by integrating an O-plasma-treated MoS_2_/MoS_2_ diode as the photosensitive layer within the gate stack. The device leverages the diode’s resistance variation with light intensity to adaptively adjust the voltage distribution across the channel, achieving a significant photoresponse. By tuning the gate voltage, the photoresponse range can be precisely controlled while maintaining high sensitivity to low-contrast targets within specific light intensity ranges. Compared to conventional photodetectors, the device demonstrates more than 1000-fold improvement in detecting low-contrast signals and significantly enhanced noise immunity. This innovation paves the way for the development of robust low-contrast target detection technologies in complex environments.

## Materials and methods

### Device fabrication

Step 1: Material preparation. Graphene, MoS_2_, and h-BN were exfoliated from bulk crystals using Scotch® tape and placed on a SiO_2_/Si substrate. Step 2: Heterostructure stacking. The h-BN as the top protective layer was picked up using a piece of propylene-carbonate (PPC), and the graphite as the source/drain electrodes, MoS_2_ as the channel, h-BN as the dielectric, MoS_2_ as the photosensitive layer, and h-BN as the bottom protective layer were then picked up in sequence. Step 3: Removing PPC. The stack was released at 130 °C on a surface of a 300-nm-thick SiO_2_ layer, which was grown on an n-doped silicon wafer (0.05-0.2 Ω·cm^−1^), followed by heating at 350 °C for 120 min in vacuum to remove the PPC. Step 4: Metal deposition. A polymethyl methacrylate (PMMA) layer (495k MW, A4, MicroChem) was spin-coated at 2000 rpm on the substrate and baked at 190 °C for 5 min, and another PMMA layer (950k MW, A2, MicroChem) was then spin-coated at 4000 rpm and baked at 190 °C for 2 min. An undercut structure was created during the electron-beam lithography and developing processes. Subsequently, the h-BN on the graphite source/drain electrodes and MoS_2_ control group gate electrodes were removed using a reactive ion etching (CHF_3_ with a flux rate of 20 sccm; O_2_ with a flux rate of 4 sccm; pressure, 2.0 Pa; power, 100 W; etching time, 1 min). Then, metal contacts for source/drain (Ti/Au: 5/50 nm) and the control group gate were formed using electron-beam evaporation and lift-off processes. Step 5: O-plasma treatment of MoS_2_. The O-plasma-treated MoS_2_ was formed using an oxygen plasma treatment (O_2_ with a flux rate of 180 sccm; power, 200 W; time, 15 min) on the MoS_2_ gate without h-BN protection. Step 6: Gate formation. Polydimethylsiloxane (PDMS) was used as the medium to transfer the graphite layer onto the O-plasma-treated MoS_2_ to form the gate.

### Characterization

Material and device characterizations were performed using an optical microscope (Nikon ECLIPSE LV100ND) and an AFM (Bruker Dimension Icon). Electrical and optoelectronic performance was measured using semiconductor analyzers (Agilent B1500A, Fs Pro, 100 kHz bandwidth), a probe station (Cascade M150), and a laser diode controller (Thorlabs ITC4001, with laser excitations of 516 nm) in a dark room at room temperature. The response time was measured using a semiconductor analyzer (Fs Pro, 100 kHz bandwidth).

### Calculation of image contrast

Image contrast of grayscale images was calculated as^36^:

$$\mathrm{Contrast}=f\left(z\right)=\mathop{\sum }\limits_{\theta }{\theta \left(i,j\right)}^{2}\times {P}_{\theta }(i,\,j)$$

where *θ*(*i*,*j*) represents the grayscale difference between two neighboring pixels, and *P*_*θ*_(*i*,*j*) denotes the probability distribution of the grayscale difference *θ*(*i*,*j*) across the image. In practical applications, the image is first expanded by adding a border of zero-grayscale pixels. Then, the grayscale differences between each pixel and its neighboring pixels (above, below, left, and right) are calculated.

### Simulation of image recognition

An ANN was used to demonstrate the robustness of our sensor in detecting low-contrast targets. The optimal parameters were determined through multiple tests, as outlined below. The Flatten layer converts the input 32 × 32 image into a one-dimensional array, allowing it to be passed to the fully connected layers. The Dense layers consist of two layers: the first with 128 neurons and the second with 64 neurons, both employing the ReLU activation function. The output layer is a Dense layer with a sigmoid activation function. The validation split is set to 0.2, and the batch size is 25. Python was used to implement the ANN for recognition tasks. The CIFAR-100 database, shown in Fig. 5b, is an open-source dataset obtained from the website (https://www.cs.toronto.edu/~kriz/cifar.html). The image pixels are 32 × 32, which meets the requirements of our hardware experimental measurements (Fig. 5b). Noise density = *N*_noise_/*N*_total_, where *N*_noise_ is the number of pixels randomly assigned to 0 or 255 (i.e., noisy pixels), and *N*_total_ is the total number of pixels in the image (32 × 32 = 1024 pixels).

### Training process of ANN

Step 1: Forward propagation. Input images were passed through the network to generate predicted outputs. Step 2: Loss calculation. The predicted outputs were compared with ground-truth labels to compute the error. Step 3: Backpropagation. The network weights were updated based on the calculated error. Step 4: Optimization. The Adam optimizer was applied to perform gradient descent and iteratively minimize the loss.

## Supplementary information

Supplementary Information

## Data Availability

The data that support the findings of this study are available from the corresponding author upon reasonable request.
