# Supplementary material for: Bioinspired phototransistor with tunable sensitivity for low-contrast target detection
Source: Light Sci Appl. 2026 Jan 1;15:12. doi: 10.1038/s41377-025-02051-1 (PMC12756283; doi:10.1038/s41377-025-02051-1)
Supplement: Supplementary file 1 — Supplementary Information [file 41377_2025_2051_MOESM1_ESM.docx]

**Supplementary Information for**

**Bioinspired phototransistor with tunable sensitivity for low-contrast target detection**

Ruyue Han, Dayu Jia, Bo Li, Shun Feng, Guoteng Zhang, Yun Sun, Zheng Han, Chi Liu, Hui-Ming Cheng & Dong-Ming Sun





**Fig. S1** Illustration of the device fabrication. **a,** Graphite (Gr), MoS_2_, and h-BN were exfoliated from their bulk crystals and were placed on to SiO_2_/Si substrate. **b,** h-BN as an encapsulation layer was picked up using a piece of propylene-carbonate (PPC). The graphite electrode, MoS_2_, h-BN, MoS_2_, h-BN were then picked up in sequence. The stack was released at 130 ℃ onto the surface of a 300-nm-thick SiO_2_ layer which was grown on a Si wafer. **c,** Heating in a vacuum at 350 °C for 120 min to remove the PPC. **d,** Metal contacts for source/drain and gate were formed using electron-beam lithography (EBL), reactive ion etching (RIE), electron-beam evaporation and lift-off processes. **e,** O-plasma treatment. **f,** Polydimethylsiloxane (PDMS) was used as the medium to transfer the top graphite gate layer onto the O-plasma-treated MoS_2_ layer.





**Fig. S2** False-color scanning electron microscope (SEM) image of the device. In the device, Gr was used for the contact and gate electrodes, MoS_2_ as the channel, h-BN as both the dielectric and protective layer, and an O-plasma-treated MoS_2_/MoS_2_ diode was inserted between the dielectric layers, serving as the photosensitive layer.

The h-BN layer plays a crucial role in balancing capacitive coupling and leakage suppression. A thinner h-BN layer can provide stronger electrostatic control but may suffer from increased leakage current, while a thicker layer improves insulation but weakens gate modulation efficiency. In our study, we optimized the thickness of the h-BN to ~20 nm to ensure both electrical stability and effective gate control. This thickness also helps to shield the underlying MoS_2_ channel from ambient influences, contributing to the overall robustness of the device.





**Fig. S3** **XPS characterization of pristine and O-plasma-treated MoS_2_.** **a-c**, XPS spectra for the pristine and O-plasma-treated MoS_2_ samples. **b,** S and Mo atomic ratio for pristine and O-plasma-treated MoS_2_ samples.

The XPS was performed to quantitatively analyze the peak positions of O 1s, Mo 3d, and S 2p, along with the elemental composition of Mo and S. As shown in Fig. S3a, the pristine MoS_2_ exhibits an O 1s peak at 532.5 eV, corresponding to adsorbed oxygen. After oxygen plasma treatment, a new O 1s peak emerges at ~531.2 eV, which is attributed to lattice oxygen, indicating the formation of chemical bonding. Comparison of the S 2p and S 2s peaks shows a significant signal decrease after plasma exposure, revealing the formation of extensive sulfur vacancies (Figs. S3b, c). Meanwhile, the Mo 3d spectrum also changes noticeably: while Mo in pristine MoS₂ mainly exists in the +4 oxidation state, part of Mo^4+^ is oxidized to Mo^6+^ after treatment, suggesting partial transformation of MoS_2_ into MoO_x_S_y_ (Fig. S3c). Quantitative analysis reveals that the S/Mo atomic ratio decreases from ~1.8 in pristine MoS_2_ to ~1.2 after plasma treatment (Fig. S3d).





**Fig. S4 High resolution transmission electron microscopy (HRTEM) and Energy Dispersive X-ray Spectroscopy (EDS) mapping from in-plane perspectives for pristine and O-plasma-treated MoS_2_.** **a**, TEM image of pristine MoS_2_. **b-d**, EDS mapping of Mo, S and O of pristine MoS_2_. **e**, TEM image of O-plasma-treated MoS_2_. **f-h**, EDS mapping of Mo, S and O of O-plasma-treated MoS_2_.

In the pristine MoS_2_, the in-plane TEM image shows clear lattice fringes (Fig. S4a), and EDS confirms a uniform distribution of Mo and S, with a small amount of adsorbed oxygen (Figs. S4b-d). After oxygen plasma treatment, the surface lattice fringes vanish, and the region becomes amorphous (Fig. S4e). While Mo and S remain uniformly distributed, a strong oxygen signal appears (Figs. S4f-h).





**Fig. S5 Raman spectra of MoS_2_ flakes before and after plasma treatment, measured using an excitation wavelength of 520 nm.**

Raman spectra show that with increasing O-plasma treatment time, the E_2g_ and A_1g_ modes gradually merge and eventually vanish, indicating thinning and eventual conversion of MoS_2_ to MoO_2_S.


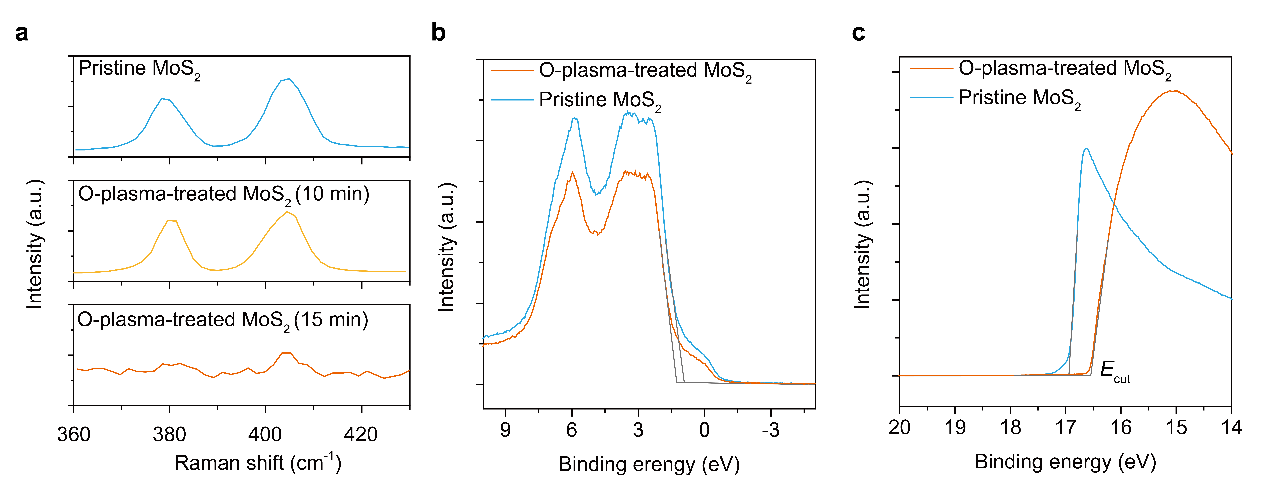


**Fig. S6 X-ray Photoelectron Spectroscopy valence band spectra of MoS_2_ flakes before and after plasma treatment.**





**Fig. S7 Band structure of pristine and O-plasma-treated MoS_2_.**


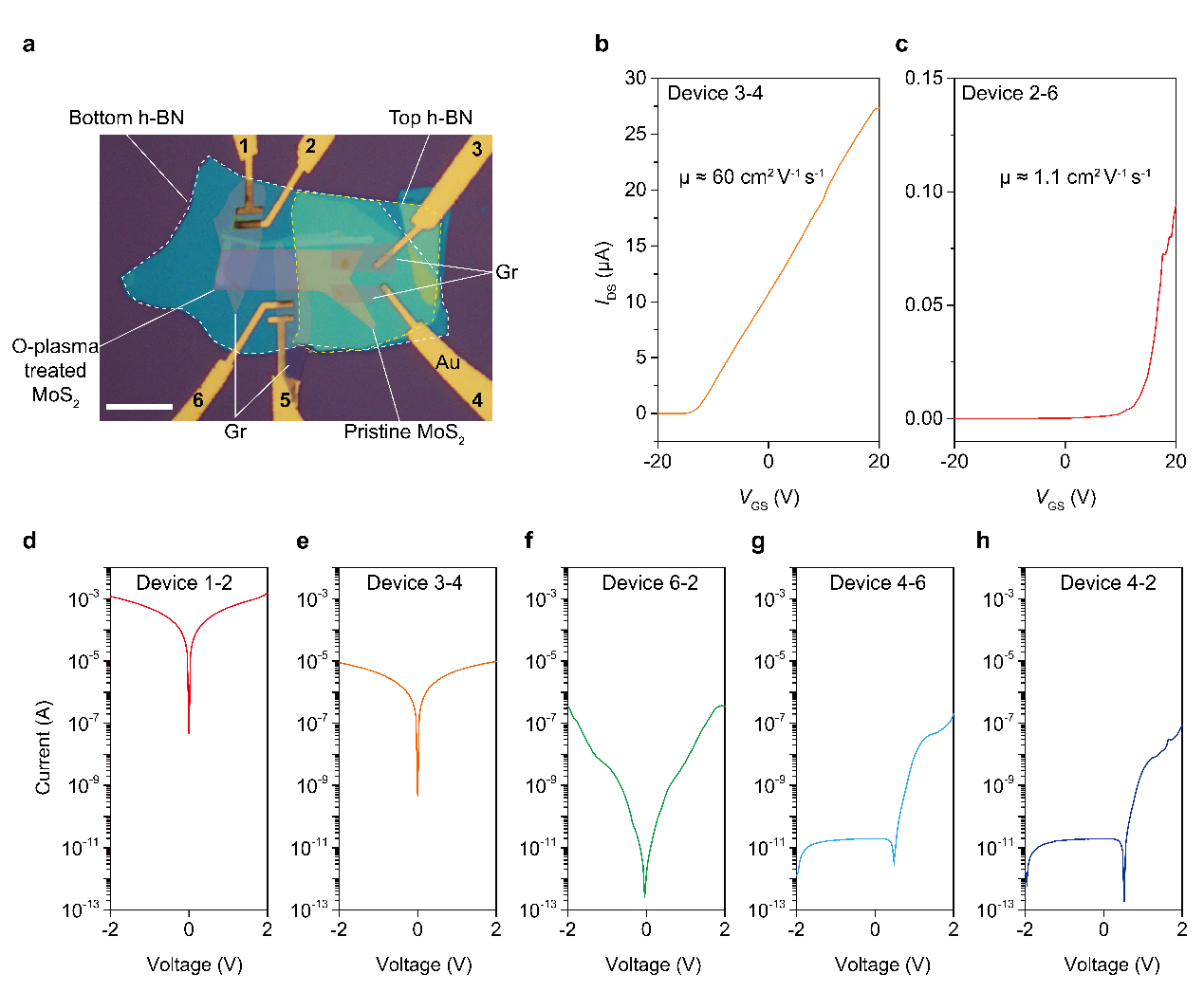


**Fig. S8 Electrical characterization of pristine and O-plasma-treated MoS_2_ and their heterojunctions. a,** Optical microscope image showing representative device structures, including Au/Gr/Au, Gr/MoS_2_/Gr, Gr/O-plasma-treated MoS_2_/Gr and Gr/MoS_2_/O-plasma-treated MoS_2_/Gr devices (Gr: graphene). Scale bar: 10 μm. **b-c**, Transfer curve of Gr/MoS_2_/Gr and Gr/O-plasma-treated MoS_2_/Gr FETs measured at a *V*_DS_ of 1 V. **d-h**, Output characteristics of Au/Gr/Au, Gr/MoS_2_/Gr, Gr/O-plasma-treated MoS_2_/Gr and Gr/MoS_2_/O-plasma-treated MoS_2_/Gr devices at a *V*_GS_ of 0 V.

To investigate the mobility, we fabricated field-effect-transistors (FETs) with graphene contacts in both pristine and plasma-treated regions on the same MoS_2_ flake (Fig. S8a). Output characteristics show that Au/Gr and Gr/MoS_2_ contacts are Ohmic (Figs. S8d, e), while Gr/O-plasma-treated MoS_2_ exhibits Schottky behavior with current >100 nA at 2 V bias (Fig. S8f). The pristine MoS_2_ FET shows a threshold voltage of -15 V and mobility ~60 cm^2^/V·s, whereas the O-plasma-treated MoS_2_ FET exhibits a threshold voltage shift to ~10 V and a reduced field-effect mobility (~1.1 cm^2^/V·s), which is attributed to the Schottky contacts (Figs. S8b, c).


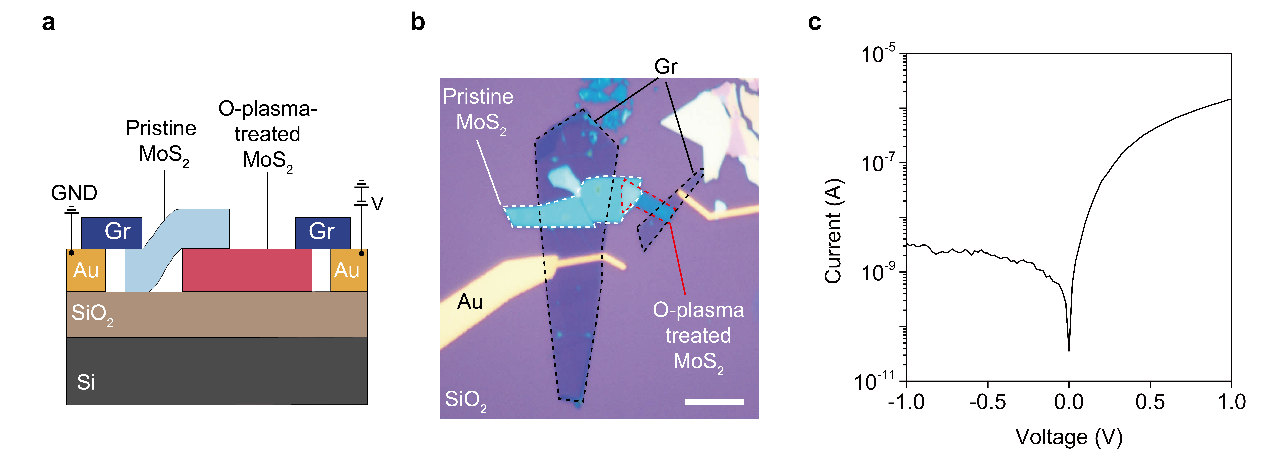


**Supplementary Fig. 9 Electrical characterization of vertical MoS_2_**/**O-plasma-treated MoS_2_ heterojunctions. a,** Device structure. **b**, Optical microscope image. **c**, Output characteristics of the diode.

To directly validate the rectifying capabilities of a vertical MoS_2_/O-plasma-treated MoS_2_ heterostructure, a vertical diode was designed and fabricated, as shown in Fig. S9a. In this structure, the top electrode is graphene, which contacts the O-plasma-treated MoS_2_ (serving as the anode), while the bottom graphene electrode contacts the pristine MoS_2_ (serving as the cathode), forming a Gr/MoS_2_/O-plasma-treated MoS_2_/Gr architecture. An optical image of the fabricated device is presented in Fig. S9b. Within a bias range of ±1 V, the device exhibits rectifying behavior with a rectification ratio approaching 10^3^, confirming that the O-plasma-treated MoS_2_ layer and pristine MoS_2_ form an asymmetric energy band alignment that supports n/n⁻ junction rectification (Fig. S9c).


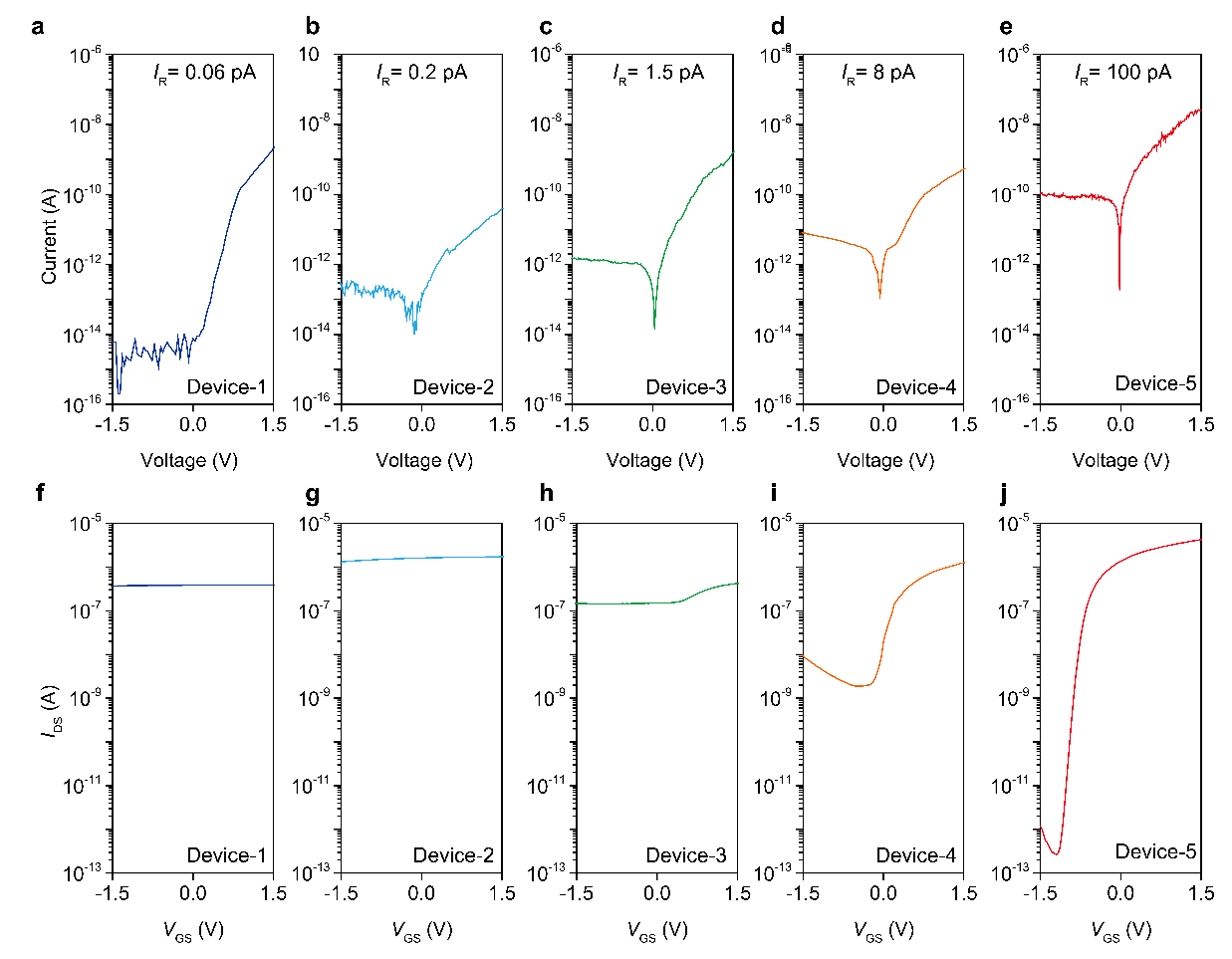


**Fig. S10 Investigation of the effect of MoS_2_/O-plasma-treated MoS_2_ heterojunction conductivity on phototransistor performance. a-e,** Output characteristics of diodes with varying reverse leakage currents. **f-j,** Transfer characteristics of phototransistors incorporating gate-integrated diodes with corresponding leakage levels.

By controlling the oxygen plasma treatment time, the MoS_2_/O-plasma-treated MoS_2_ junction gradually transforms from a vertical to a lateral structure, thereby modulating the reverse leakage current of the junction. When the reverse leakage current of the diode is low (< 1 pA), the phototransistor exhibits an on/off ratio below 2, indicating that the MoS_2_/O-plasma-treated MoS_2_ junction effectively blocks gate current (Figs. S10a-c, f-h). As the reverse leakage current of the MoS_2_/O-plasma-treated MoS_2_ junction increases, the on/off ratio of our transistor increases. When the reverse current exceeds 10 pA, our transistor behaves as a conventional transistor (Figs. S10d, e, I, j).





**Fig S11 Investigation of the effect of temperature on MoS_2_/O-plasma-treated MoS_2_ heterojunction conductivity and phototransistor performance. a,** Output characteristics of diodes with varying temperature. **b,** Photoresponse behavior of the device under a 516-nm light pulse (light intensity: 6650 μW cm^-2^, pulse width: 100 ms) and simultaneous gate voltage pulses of -3 V (pulse width 100 ms) at *V*_DS_ = 0.1 V.

As temperature decreases, the reverse bias current of the diode significantly drops, indicating a thermionic emission mechanism at the heterointerface (Fig. S11a). Correspondingly, the photoresponse weakens due to the reduced reverse current (Fig. S11b).





**Fig. S12 Photoresponse behavior comparison between the tunable-sensitivity phototransistor and the MoS_2_/O-plasma-treated MoS_2_ heterojunction.** **a,** Current ratio of the tunable-sensitivity phototransistor under 516 nm illumination at *V*_DS_ = 0.1 V. **b,** Output characteristics of the MoS_2_/O-plasma-treated MoS_2_ photodiode under different illumination intensities.





**Fig. S13 Schematic illustrations and corresponding transfer characteristics under different illumination conditions to investigate the device photoresponse.** **a-c,** Schematics showing illumination on (a) only the MoS_2_/O-plasma-treated MoS_2_ heterojunction region, (b) both the channel and junction regions simultaneously, and (c) only the MoS_2_ channel region. **d-f,** Transfer curves measured in dark and under illumination corresponding to each scenario in (a-c), respectively.

A series of comparative experiments was designed to systematically investigate how different illumination positions affect the photoresponse of the device (Fig. S13). Specifically, three illumination conditions were studied: in Supplementary Fig. 13a, light irradiates only the MoS_2_/O-plasma-treated MoS_2_ heterojunction region; in Fig. S13b, light irradiates both the channel and junction regions simultaneously; and in Fig. S13c, light irradiates only the MoS_2_ channel region. The experimental results reveal that when the light is confined to the junction region, the device exhibits a pronounced negative photoresponse with an on/off ratio approaching 10^6^ (Fig. S13d). In contrast, simultaneous illumination of both the junction and channel leads to a significant reduction in the photo-switching ratio by more than one order of magnitude (Fig. S13e). To further assess the intrinsic photoresponse of the MoS_2_ channel, standalone MoS_2_ field-effect transistors were fabricated and tested, showing a positive photoresponse with a switching ratio greater than 10 under channel illumination, confirming the photoactivity of the MoS_2_ channel (Fig. S13f).

Based on these findings, we conclude that simultaneous illumination induces competing photoresponses: the positive photocurrent from the MoS_2_ channel partially offsets the negative photogating effect from the junction, reducing the overall switching ratio. Although this interference is minor, it was minimized in our measurements by carefully controlling spot size and illumination position to ensure the heterojunction response dominates. For future applications, we recommend encapsulating the channel region with an opaque material to fully suppress its photoresponse and improve device performance consistency.



**Fig. S14 Photoresponse behavior of the device under a 516-nm light pulse with different light intensity (*P*_in_) (pulse width 100 ms) and with a gate voltage (*V*_GS_) pulse (pulse width 100 ms) at *V*_DS_ = 0.1 V.** **a**, 0 to -7 V, **b**, 0 to -5 V, **c**, 0 to -3 V, **d**, 0 to -1 V.

The observed residual current is primarily attributed to the delayed release of photo-generated carriers from trap states at the MoS_2_/h-BN interface. During illumination, trap sites—such as surface states or structural defects—can capture carriers, and upon cessation of illumination, these trapped carriers are released slowly, resulting in a gradual decay of the photocurrent.

Regarding the impact on imaging applications, the influence of residual current is limited. In practical machine vision tasks, image recognition is performed based on the relative modulation of current in response to light, rather than the absolute current level. Therefore, slight current offsets introduced by residual trapping have negligible effect on the system’s ability to extract features or classify images. Moreover, background normalization and correction procedures should be applied during the data preprocessing stage to mitigate such artifacts.





**Fig. S15 Photoresponse behavior of a conventional MoS_2_ phototransistor under a 516-nm light with different light intensity (*P*_in_) at *V*_DS_ = 0.1 V.**

As the light intensity increases, the responsivity of the tunable-sensitivity phototransistor gradually decreases. In contrast, the conventional MoS_2_ phototransistor lacked this characteristic. It should be noted that in a conventional transistor, the carrier density increases linearly with light intensity, typically resulting in a near-constant responsivity. However, the tunable-sensitivity phototransistor keeps in the on-state in the dark, and when it is under light, illumination redistributes the gate voltage such that a larger portion is applied across the channel, thereby turning the transistor off. Therefore, the responsivity (*R*) is defined as *R* = (*I*_Dark_-*I*_Light_) *P*_in_^-1^, where *I*_Dark_ is the current measured in the dark, and *I*_Light_ is the current under illumination. This negative photoresponse behavior is fundamentally different from that of a traditional phototransistor. According to this equation, when light power increases, *I*_Dark_ keeps a relative large constant, and *I*_Light_ gradually decreases, *I*_Dark_-*I*_Light_ increase, however, cannot catch up the increase speed of *P*_in_, resulting in a decreased responsivity. In short, in a traditional transistor *I*_Light_ increases with *P*_in_, and in our transistor, *I*_Light_ decreases with *P*_in_, therefore causing a difference behavior of responsivity. This nonlinearity is reminiscent of the “light adaptation” behavior of the human eye, reflecting bio-inspired visual adaptability.





**Fig. S16 Statistical analysis of the optoelectronic performance of the phototransistor array.** **a,** Distribution of the current of device in the dark and under the illumination (516-nm light, 2.3 mW cm^-2^). **b,** Responsivity statistics. **c,** Dark-to-light current ratio statistics.

As shown in Fig. S16a, the dark-state current of the arrayed devices is centered around ~5×10^-7^ A, while under illumination at 2.3 mW cm^-2^, the photocurrent primarily falls within the range of 10^-11^ to 10^-10^ A. Further analysis reveals that the responsivity of the devices is predominantly distributed between 4×10^3^ and 5×10^3^ A W^-1^ (Fig. S16b), with a consistent photo-to-dark current ratio of approximately 10^4^ (Fig. S16c), indicating excellent uniformity across the array.


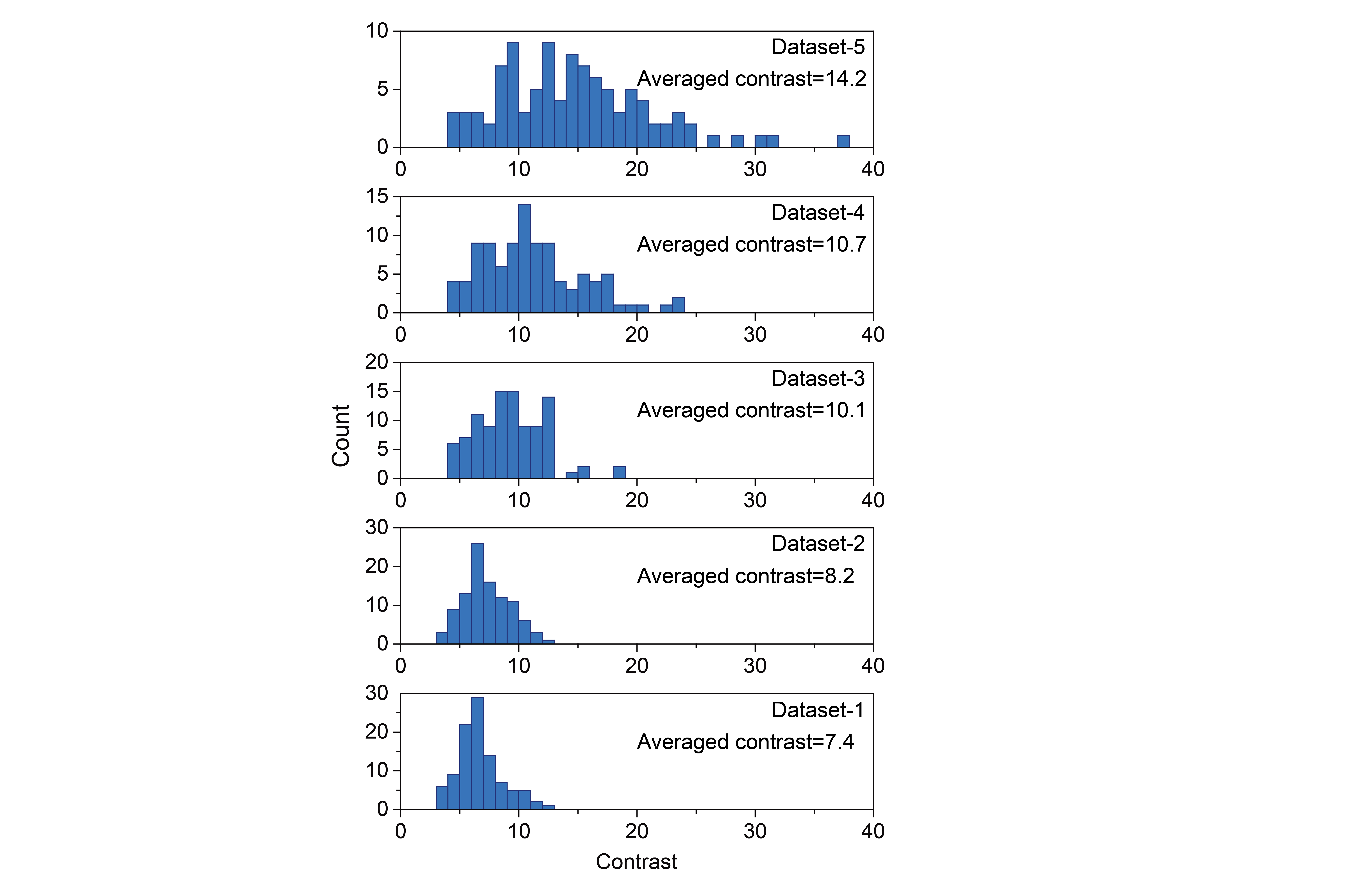


**Fig. S17 Distribution of image contrast and the calculated averaged contrasts for the extended CIFAR100 dataset.**


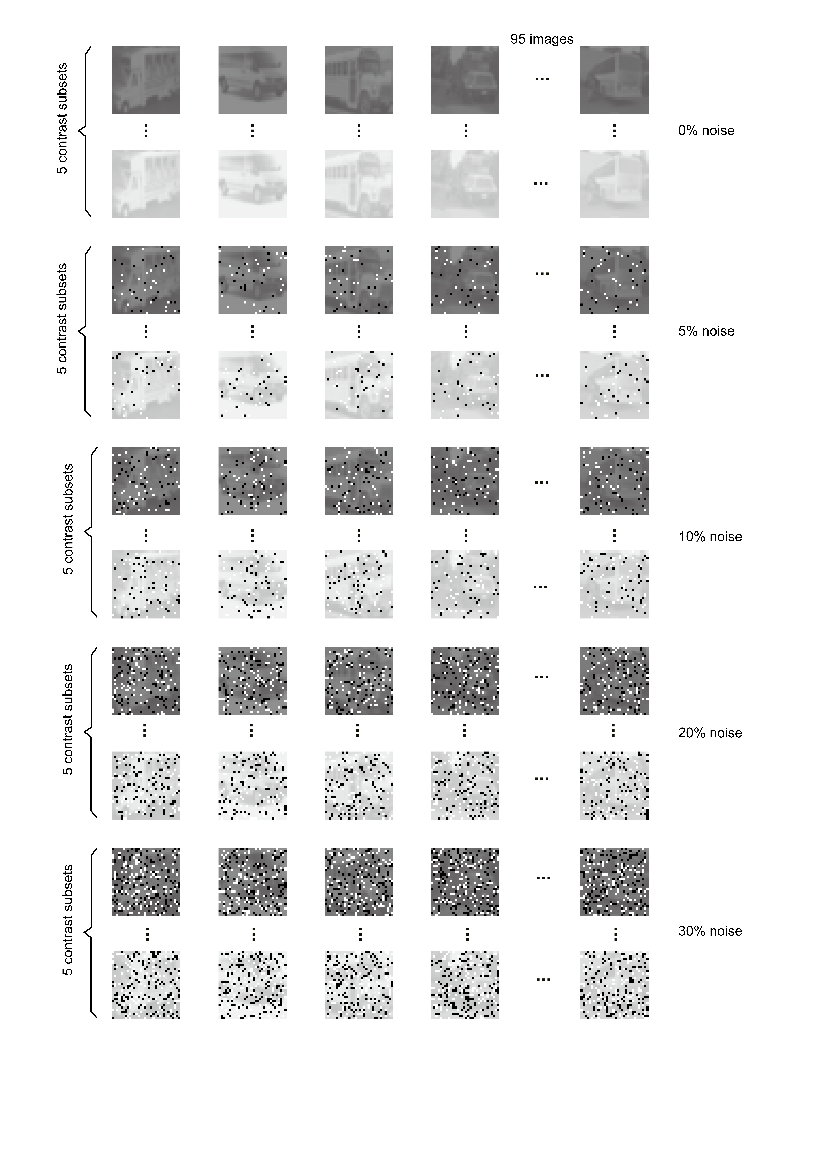


**Fig. S18 Low-contrast and noisy test datasets.**

**Table S1 Performance comparison of the proposed phototransistor with previously reported gate-tunable phototransistors for image contrast enhancement.**


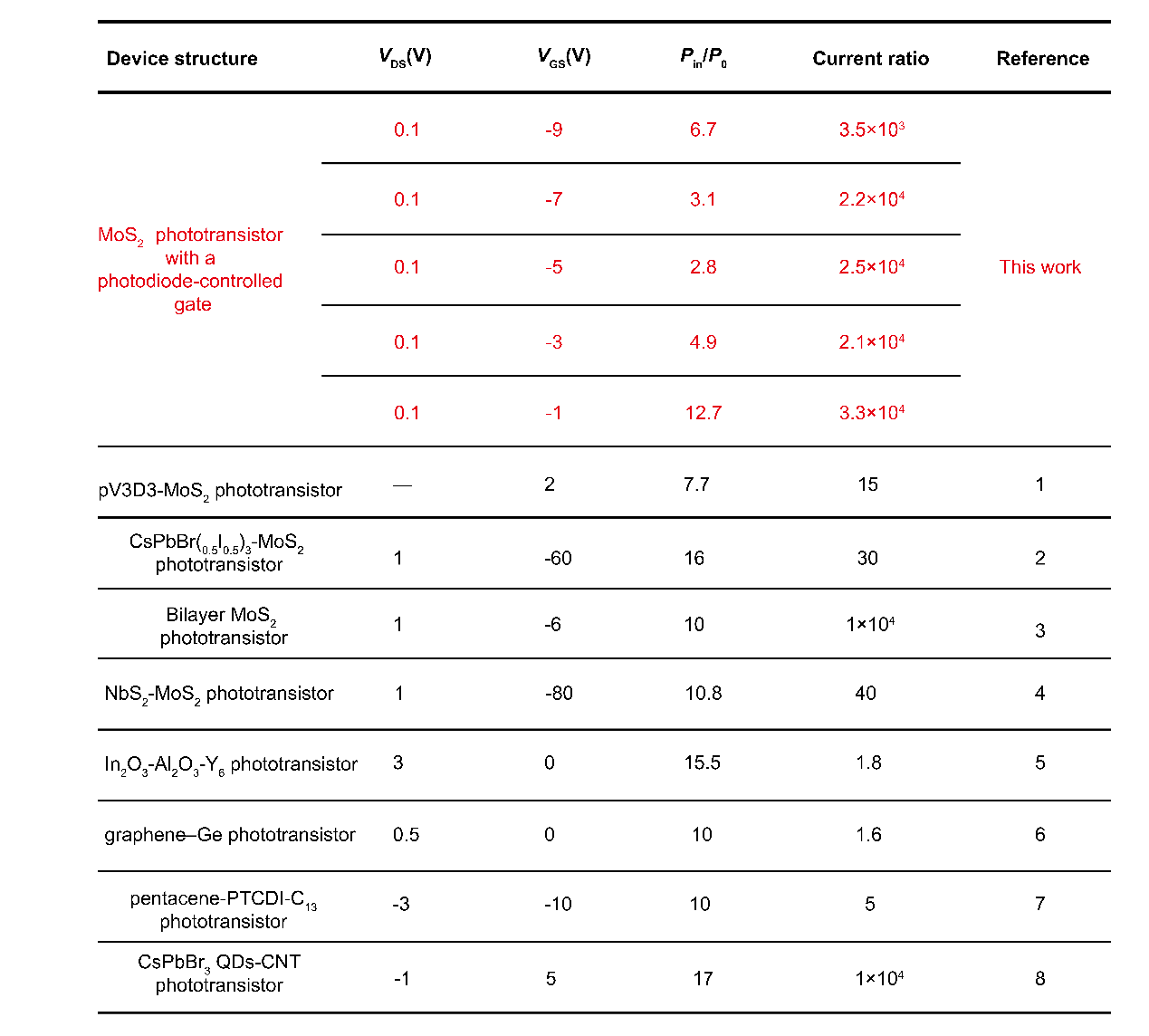


Current ratio (tunable-sensitivity phototransistor: *I*_0_/*I*_light_; conventional phototransistor: *I*_light_/*I*_0_); light intensity ratio (*P*_in_/*P*_0_), where *P*_0_ represents the minimum detectable light intensity, and *I*_0_ is the corresponding current.

**Table S2 Optimal *V*_GS_ selection under different light intensities.**





In real-world low-contrast environments, the system selects the optimal *V*_GS_ based on the actual light intensity received by the phototransistor. As shown in Fig. 4b in the manuscript, each *V*_GS_ corresponds to a specific light intensity range where the device exhibits the highest photoresponse sensitivity, i.e., the steepest slope in the *I*_DS_-*P*_in_ curve. The optimal *V*_GS_ values corresponding to different light intensity intervals are listed in Table S2.

In practical applications, if the ambient illumination range can be estimated in advance, the system directly selects the corresponding optimal *V*_GS_ for recognition. In unfamiliar environments where the illumination is unknown, an initial *V*_GS_ may be selected based on a default value or real-time photocurrent sampling. The system can then dynamically adjust *V*_GS_ during the recognition process based on feedback to enhance accuracy and stability.

**Reference:**

1. Kwon, J. et al. In-sensor multilevel image adjustment for high-clarity contour extraction using adjustable synaptic phototransistors. *Sci. Adv.* **11**, eadt6527(2025).
2. Hong, S. et al. Sensory Adaptation and Neuromorphic Phototransistors Based on CsPb(Br1–xIx)_3_ Perovskite and MoS2 Hybrid Structure. *ACS Nano* **14**, 9796-9806 (2020).
3. Liao, F. et al. Bioinspired in-sensor visual adaptation for accurate perception. *Nature Electron.* **5**, 84–91 (2022).
4. Huang, PY. et al. Neuro-inspired optical sensor array for high-accuracy static image recognition and dynamic trace extraction. *Nature Commun.* **14**, 6736 (2023).
5. Li, D. et al. Bidirectionally photoresponsive optoelectronic transistors with dual photogates for all‐optical‐configured neuromorphic vision. *Adv. Funct. Mater*. **33,** 2303198 (2023).
6. Yang, Y. et al. In-sensor dynamic computing for intelligent machine vision. *Nature Electron.* **7**, 225–233 (2024).
7. Wang, Y. et al. Dual-adaptive heterojunction synaptic transistors for efficient machine vision in harsh lighting conditions. *Adv. Mater.* **36**, 2404160 (2024).
8. Zhu, Q.-B. et al. A flexible ultrasensitive optoelectronic sensor array for neuromorphic vision systems. *Nature Commun.* **12**, 1798 (2021).
